# Expansion of CD8+CD57+ T Cells in an Immunocompetent Patient with Acute Toxoplasmosis

**DOI:** 10.1155/2009/173439

**Published:** 2009-09-14

**Authors:** R. García-Muñoz, P. Rodríguez-Otero, A. Galar, J. Merino, J. J. Beunza, J. A. Páramo, R. Lecumberri

**Affiliations:** ^1^Department of Hematology, Hospital San Pedro, Logroño, La Rioja 26006, Spain; ^2^Department of Hematology, University Clinic, University of Navarra, 31008 Navarra, Spain; ^3^Department of Microbiology, University Clinic, University of Navarra, 31008 Navarra, Spain; ^4^Department of Immunology, University Clinic, University of Navarra, 31008 Navarra, Spain; ^5^Department of Preventive Medicine and Public Health, University of Navarra, 31008 Navarra, Spain

## Abstract

CD57+ T cells increase in several viral infections like cytomegalovirus, herpesvirus, parvovirus, HIV and hepatitis C virus and are associated with several clinical conditions related to immune dysfunction and ageing. We report for the first time an expansion of CD8+ CD57+ T cells in a young patient with an acute infection with Toxoplasma gondii. Our report supports the concept that CD8+ CD57+ T cells could be important in the control of chronic phase of intracellular microorganisms and that the high numbers of these cells may reflect the continuing survey of the immune system, searching for parasite proliferation in the tissues.

## 1. Introduction

*Toxoplasma gondii*, an obligate intracellular protozoan, can invade and replicate in almost any nucleated host cell, being cats and their pray the definitive hosts. Oocysts shed in cat feces can infect a wide range of animals including humans. Infection occurs by ingestion of parasite-cyst-contaminated food or water; cysts rupture in the host, and the released parasites actively enter host cells [1]. Certain individuals are at high risk for severe disease, especially congenitally infected fetuses and newborns and immunologically impaired individuals [2]. Infection in immunocompetent hosts is usually asymptomatic and self-limited, and it does not normally require therapy [2]. Individuals infected with *Toxoplasma gondii* require a powerful immune response to contain dissemination of the parasite, resulting in a strong and persistent T-helper-1 (Th1) response characterised by production of proinflammatory cytokines including IL-12, INF-*γ*, and TNF-*α* [3]. Human CD4+ and CD8+ T lymphocytes are cytotoxic to Toxoplasma gondii-infected cells [4]. However, the possible implication of CD8+CD57+ T cells in the control of acute infection and the tissues survey in the chronic phase has never been described. We report a patient with acute toxoplasmosis with increased levels of CD8+CD57+ T cells in peripheral blood.

## 2. Case Report

**Figure 1:**
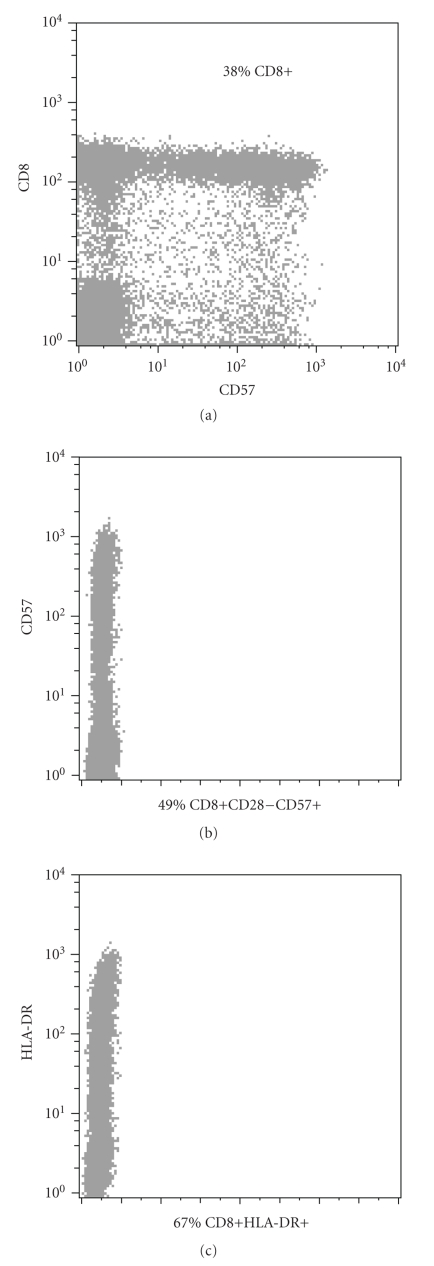
Increased number of CD8+ T cells with a high grade of activation (HLA-DR) and replicative senescence (CD57+).

A 20-year-old man presented with a 3-week history of fatigue and cervical lymphadenophaty. Chest X-ray was normal, but reactive laterocervical and jugulodigastric lymphadenophaties were found in cervical ultrasound. At presentation, peripheral blood count revealed a white blood cell count (WBC) of 5.9 × 10^9^/L with 58.9% lymphocytes (3.5 × 10^9^/L) and absolute neutrophil count (ANC) of 29.7% (1.75 × 10^9^/L), and blood smear demonstrated the presence of numerous stimulated lymphocytes with intracytoplasmatic granules. Screening for infectious disease was performed including HIV, Epstein-Barr virus, cytomegalovirus, and *Toxoplasma gondii*. All results were negative except for positive IgM and IgG enzyme-linked immunosorbent assay to *Toxoplasma gondii*; the weakly IgG avidity test (low avidity) confirmed recent acquired infection. Flow cytometry analysis revealed an increase in the number of CD8+ T cells with a high grade of activation (67% expressed HLA-DR) and replicative senescence (49% expressed CD57); see Figure 1. Normal levels of CD4+ T cells and B cells were also observed (Table 1). The patient is currently asymptomatic and without treatment, with few nontender and discrete cervical lymph nodes.

## 3. Discussion

CD57+ T lymphocytes are virtually absent at birth [5] and progressively increasing with age [6]. CD8+CD57+ T cells increases in chronic immune activation states and in infectious diseases like HIV [7], tuberculosis [8], and some virus, particularly cytomegalovirus [9, 10]. However, increased numbers of these cells in toxoplasmosis have never been reported before. Interestingly, INF-gamma production is directly correlated with CD8+CD28-CD57+ T cells and age [11]. More over, IFN-gamma is crucial in protective immunity against *Toxoplasma gondii* infection, and CD8+CD57+ T cells can proliferate and produce high amounts of INF-gamma and IL-5 [12]. Both cytokines are related with a protective role against *Toxoplasma gondii* infection [13]. Taken together, is possible that CD8+CD57+ T cells could prevent the reactivation of old intruders that cannot be cleared in the young age, in particular CMV [9] and *Toxoplasma gondii* infections. We suggest that CD8+CD57+ T prevent the reactivation of Toxoplasma gondii in a similar manner that CD8+CD57+ T cells can control CMV reactivation in elderly individuals.

## Figures and Tables

**Table 1:** T cells, B cells, and NK cells counts by flow cytometry in whole peripheral blood.

	% whole blood cells	Cells/*μ*L	Normal range cells/*μ*L
T cells	54	3186	933–2491
CD4+ T cells	15	885	370–1468
CD8+ T cells	38	2242	183–799
NK cells	6.7	395	60–495
B cells	2	118	65–595
